# Oxygen-Deficient TiO_2_ Aerogel for Enhanced Photocatalytic Performance

**DOI:** 10.3390/gels12050370

**Published:** 2026-04-28

**Authors:** Haochen Jiao, Wenxuan Wang, Cong Li, Yizhe Wang, Meng Yuan, Yudong Li, Daxin Liang

**Affiliations:** Key Laboratory of Bio-Based Material Science and Technology (Ministry of Education), Northeast Forestry University, Harbin 150040, China; m13356387671@163.com (H.J.); wxwang@nefu.edu.cn (W.W.); lc18846103631@163.com (C.L.); wyz20010613@163.com (Y.W.); scymwy@163.com (M.Y.)

**Keywords:** TiO_2_, oxygen vacancies, porous structure, photocatalytic degradation

## Abstract

Low charge-separation efficiency is a major factor limiting the photoelectric conversion performance of TiO_2_. In this work, oxygen-vacancy-rich porous TiO_2_ gel photocatalyst was successfully fabricated. The as-prepared material exhibits a three-dimensional interconnected hierarchical porous architecture with a specific surface area of 62.9 m^2^ g^−1^. EPR and XPS analyses confirmed the presence of Ti^3+^ defects and oxygen vacancies, which effectively increase the electron density and facilitate the separation and migration of photogenerated charge carriers. The results demonstrated excellent photocatalytic activity, with over 85% of RhB degraded within 50 min under light irradiation. In addition, its photocatalytic performance was further investigated by photocatalytic hydrogen evolution, and the hydrogen production rate reached 850.6 μmol·g^−1^ h^−1^. The enhanced photocatalytic performance can be mainly attributed to the synergistic effect of the hierarchical porous structure and oxygen vacancies. Specifically, the hierarchical porous structure improves mass transfer and provides abundant active sites, while oxygen vacancies modulate the electronic structure and promote charge separation, thereby significantly enhancing the catalytic activity. This work provides an effective strategy for improving the photoelectric conversion performance of TiO_2_ and offers theoretical guidance as well as experimental support for the defect engineering and structural design of TiO_2_-based photocatalytic materials.

## 1. Introduction

Hydrogen, owing to its high energy density, clean combustion products, and sustainable recyclability, is widely regarded as an important secondary energy source for replacing fossil fuels and alleviating the energy crisis and environmental pollution [1,2,3]. Solar-driven photocatalytic water splitting over semiconductors is considered an ideal approach for achieving green hydrogen production and solar energy conversion [4,5,6,7]. Among various semiconductor photocatalysts, TiO_2_ has become one of the most extensively studied and representative materials in the field of photocatalytic hydrogen production because of its high chemical stability, low cost, non-toxicity, and excellent resistance to photocorrosion [8,9]. However, the practical application of TiO_2_ is still restricted by several factors [10,11,12]. First, TiO_2_ possesses a wide band gap and can only respond to ultraviolet light, which accounts for only a very small fraction of the solar spectrum, resulting in low solar energy utilization efficiency [10]. Second, the photogenerated electron–hole pairs in TiO_2_ tend to recombine rapidly, which severely limits the separation and transport efficiency of charge carriers [12].

To improve the photoelectric conversion efficiency of TiO_2_, researchers initially introduced various cation dopants [13,14,15] (such as Nd, Sb, and Ag) or anion dopants [16,17,18] (such as S, C, and N) to enhance light absorption. However, the high cost of metal cations, the complexity of the doping process, and the limited catalytic performance achieved by anion doping have restricted the practical application of doping technology in TiO_2_ photocatalysis. In recent years, defect engineering has been widely regarded as an effective strategy for tuning the physicochemical properties and surface actives sites of semiconductor photocatalysts. In particular, oxygen-vacancy-rich TiO_2_ (Ti^3+^) materials have been widely used in photocatalysis because of their excellent catalytic performance [19,20,21,22,23,24]. Oxygen vacancies are generally regarded as crucial defect sites for regulating the electronic structure and surface properties of semiconductor photocatalysts, increasing the carrier density, facilitating the separation and migration of photogenerated charge carriers, thereby improve interfacial charge-transfer efficiency [20,21,22]. In addition, oxygen vacancies can act as active centers for the adsorption and activation of reactant molecules, which is highly beneficial for enhancing the overall photocatalytic performance [23]. However, due to the limited specific surface area of conventional TiO_2_, most oxygen vacancies are confined to exposed surface active sites, resulting in unsatisfactory catalytic performance. Moreover, a low specific surface area often leads to a high recombination rate of photogenerated electrons and holes.

Therefore, the construction of porous architectures is of great significance for improving the catalytic performance of defect-engineered TiO_2_ systems. Various porous TiO_2_ materials have been prepared using soft and hard templates, which can effectively enhance light absorption and photocatalytic efficiency. More importantly, porous structures are also beneficial for defect engineering, as they facilitate the formation and exposure of defect sites and can increase the concentration of oxygen vacancies [25,26,27,28]. In particular, porous TiO_2_ derived from a TiO_2_ gel precursor provides a promising platform for integrating structural regulation with defect modulation.

To improve the photocatalytic hydrogen evolution performance of TiO_2_, porous oxygen-vacancy-rich TiO_2_ microspheres gel were constructed through a gel-assisted strategy. TiO_2_ gel precursor was first synthesized and then converted into porous TiO_2_ microspheres gel enriched with oxygen vacancies. The enhanced photocatalytic activity of the gel-derived porous TiO_2_ originates from the synergistic effects of defect engineering and porous structural regulation. Oxygen vacancies introduce defect states into the band gap of TiO_2_ and modulate its electronic structure, thereby extending the light-response range and improving visible-light absorption. In addition, these defects serve as electron-trapping centers, which inhibit the recombination of photogenerated electron–hole pairs and facilitate charge separation. The coexistence of Ti^3+^ species further increases electron density and electrical conductivity, lowers charge-transfer resistance, and accelerates electron migration from the bulk to the surface. Meanwhile, the kinetics of hydrogen evolution are significantly improved, Benefiting from the synergistic optimization of light harvesting, charge separation, charge transport, and surface reaction processes.

## 2. Results and Discussion

### 2.1. Morphology and Composition Analysis

Tetrabutyl titanate (TBT) was selected as the titanium precursor. Compared with TiCl_3_ and TiCl_4_, TBT is easier to control during the reaction process and is less likely to undergo rapid hydrolysis or generate large amounts of acidic by-products. Therefore, the reaction process involving TBT is relatively mild and safe [29,30,31]. IRA-900 was employed as a soft template and fully impregnated with TBT pre-hydrolyzed in hydrochloric acid solution. The resulting TBT/IRA-900 composite was then aged at 60 °C, leading to the formation of an acidic TiO_2_ sol via flocculation. After drying, a gray porous TiO_2_ gel was obtained and subsequently subjected to calcination. The hydrolysis and polycondensation reactions of TBT in hydrochloric acid solution are shown in Equations (1)–(3) [32,33].

Ti(OC_4_H_9_)_4_ + nH_2_O → Ti(OC_4_H_9_)_4−n_(OH)_n_ + nC_4_H_9_OH (1)

Ti(OC_4_H9)_4−n_(OH)_n_ + Ti(OC_4_H_9_)_4−n_(OH)_n_ → (C_4_H_9_O)_4−n_Ti-O-Ti(OC_4_H_9_)_4−n_ + nH_2_O (2)

Ti(OC_4_H_9_)_4−n_(OH)n + Ti(OC_4_H_9_)_4_ → (C_4_H_9_O)_4−n_ × Ti-O-Ti(OC_4_H_9_)_4−n_ + nC_4_H_9_OH (3)

**Figure 1:**
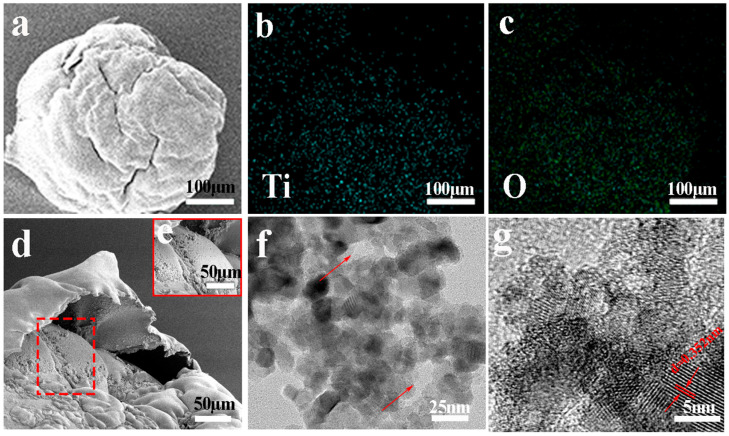
(**a**–**c**) SEM and EDS of porous TiO_2-X_-550, where (**b**)Ti spectrum and (**c**) O spectrum, (**d**,**e**) SEM of the fractured surface of TiO_2-X_-550, and (**f**,**g**) TEM of TiO_2-X_-550. The arrows indicate the channels, and d = 0.352 nm represents the lattice spacing.

The morphology and elemental distribution of porous TiO_2-X_-550 were characterized by SEM and EDX mapping. As shown in Figure 1a, porous TiO_2-X_-550 exhibits an approximately spherical morphology with a wrinkled surface, which is beneficial for enhanced light absorption [34]. As shown in Figure 1d,e, the fractured surface of porous TiO_2-X_-550 exhibits a distinct internal porous structure, which is beneficial for light absorption, mass transfer, and the transport of reactive species. The EDS mapping images in Figure 1b,c confirm that the sample consists solely of Ti and O elements, indicating the successful formation of TiO_2_. In addition, the corresponding EDAX spectrum is presented in Figure S1 (Supplementary Materials), further verifying the elemental composition of porous TiO_2-X_-550. TEM analysis further reveals that porous TiO_2-X_-550 possesses a hierarchical pore structure with pore sizes of approximately 5–30 nm, and the pores are continuous and interconnected (Figure 1f). In addition, the HRTEM image in Figure 1g shows lattice fringes with d-spacings of 0.352 nm, which can be assigned to the (101) planes of anatase TiO_2_, respectively [35,36].

**Figure 2:**
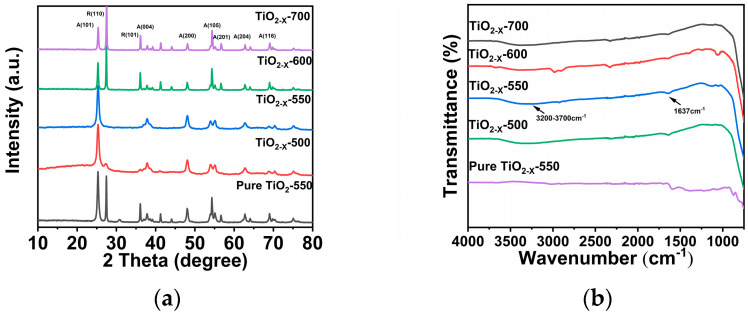
(**a**) XRD patterns of porous TiO_2−X_ calcined at different temperatures; (**b**) FT−IR spectra of porous TiO_2−X_ calcined at different temperatures.

The crystal structures of the TiO_2-X_ samples prepared at different calcination temperatures were examined by XRD (Figure 2a). The diffraction peaks of TiO_2-X_-550 at 25.3°, 37.8°, 48.0°, 53.9°, 54.9°, 62.9°, and 68.8° are assigned to the (101), (004), (200), (105), (201), (204), and (116) planes of anatase TiO_2_, respectively, indicating that the sample predominantly consists of the anatase phase [36,37]. The XRD patterns clearly reveal the presence of the anatase TiO_2_ phase in all samples. Compared with TiO_2-X_-500 and TiO_2-X_-550, the samples prepared under the other calcination conditions show, in addition to the characteristic anatase diffraction peaks, two extra peaks located at 27.41° and 36.09°, corresponding to the (110) and (101) planes of rutile TiO_2_, respectively. This indicates that partial phase transformation from anatase to rutile occurred under these calcination conditions [38]. These results indicate that a portion of anatase TiO_2_ was transformed into rutile TiO_2_, which can be attributed to the anatase-to-rutile phase transition of TiO_2_ occurring within the temperature range of 550–800 °C. Therefore, the samples calcined below 550 °C are expected to retain more lattice defects, which can serve as charge-trapping sites and facilitate the separation of photogenerated electron–hole pairs, thereby contributing to enhanced photocatalytic performance. According to the Scherrer equation: D = K⋅λ/β⋅cosθ: where D is the average crystallite size (nm), K is the Scherrer constant (typically 0.89–0.94, usually taken as 0.9), λ is the X-ray wavelength, β is the full width at half maximum (FWHM) of the diffraction peak in radians, and θ is the Bragg angle, the crystallite sizes of TiO_2-X_-550 were calculated to be 7.7–16.7 nm (Table S1 in Supplementary Materials). Interestingly, the crystallite size corresponding to the (101) plane first increased and then decreased as the calcination temperature increased. Among all the samples, TiO_2-X_-550 exhibited the largest crystallite size along the (101) plane. In addition, its diffraction peak remained relatively broad, suggesting a high degree of lattice disorder and/or microstrain, which is favorable for defect formation in the material. To further investigate the molecular structure and surface chemical composition of TiO_2-X_ prepared under different calcination conditions, Fourier transform infrared (FTIR) spectroscopy was carried out (Figure 2b). The absorption band at 1637 cm^−1^ is assigned to the bending vibration of adsorbed water molecules, while the broad band observed at 3200–3700 cm^−1^ corresponds to the stretching vibration of surface hydroxyl groups. These results indicate the presence of abundant hydroxyl species and adsorbed water on the TiO_2_ surface [39].

**Figure 3:**
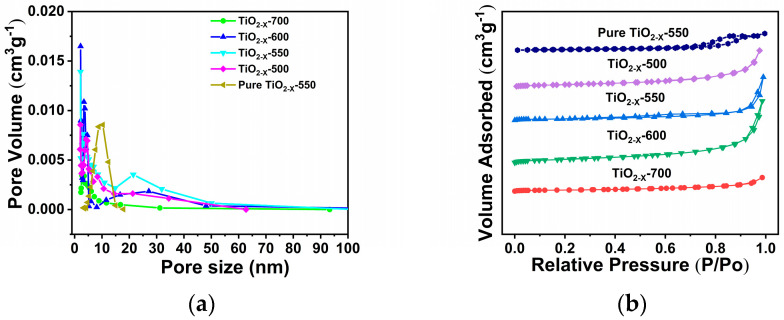
(**a**) Pore size distribution and (**b**) N_2_ adsorption–desorption isotherms of porous TiO_2−X_ at different calcination temperatures.

The specific surface areas and pore structures of all samples were investigated by nitrogen adsorption–desorption measurements. As shown in Figure 3a, TiO_2-X_-500, TiO_2-X_-550, and TiO_2-X_-600 exhibited pronounced H_3_-type hysteresis loops in the relatively high-pressure region (P/P_0_), indicating the presence of abundant mesopores and hierarchical pore channels within the materials [40,41,42,43]. In contrast, the other samples did not exhibit obvious H_3_ hysteresis loops in the adsorption–desorption isotherms. The pore-size distribution curves in Figure 3b further show that TiO_2-X_-500, TiO_2-X_-550, and TiO_2-X_-600 possess pores distributed in the range of 2–60 nm, confirming the successful formation of hierarchical porous structures, summarized in Table S2, TiO_2-X_-550 exhibits the largest specific surface area, reaching 62.9 m^2^ g^−1^, In general, the specific surface area tends to decrease with increasing calcination temperature, which can be attributed to particle growth and partial pore collapse during calcination. To further clarify the three-dimensional pore structure of TiO_2-X_-550, Micro-CT analysis was performed. The results indicate that TiO_2-X_-550 possesses a highly porous framework with predominantly interconnected open pores, which is in good agreement with the BET results, summarized in Figure S2 and Table S3. Such a pore architecture is favorable for reactant diffusion and active-site accessibility.

**Figure 4:**
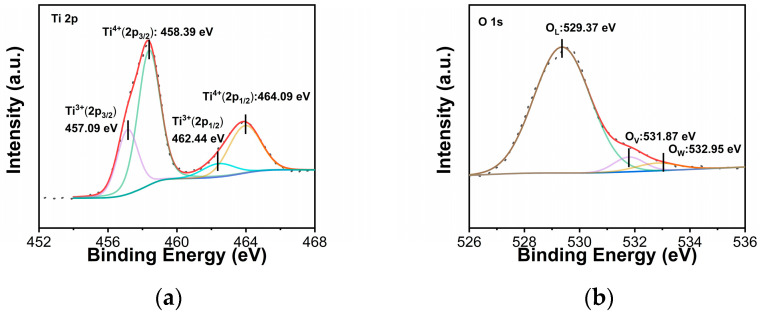
(**a**) XPS spectra of porous TiO_2−X_-550, in which Ti 2p (**a**) and O 1s (**b**).

The chemical valence state and surface composition of porous TiO_2-X_-550 were analyzed by XPS. The corresponding XPS survey spectrum is presented in Figure S3, confirming the presence of Ti and O elements in the sample. Figure 4a shows that the peaks at 457.09 eV and 462.44 eV were assigned to Ti 2p_3/2_ and Ti 2p_1/2_ of porous TiO_2-X_-550, which be attributed to Ti^3+^ in the sample, while the peaks at 458.39 eV and 464.09 eV were assigned to Ti^4+^ in the sample [44]. In Figure 4b, the O1s spectrum indicates that the peaks at 529.37 eV, 531.87 eV and 532.95 eV have obvious peaks, which correspond to lattice oxygen (O_L_), oxygen atoms near the oxygen vacancy (O_V_) and chemically adsorbed water molecules (O_W_) formed by the adsorbed water molecules [42]. In Figure S4, the EPR spectra show that all samples exhibit a distinct resonance signal around 3428–3432 G, indicating the presence of a certain concentration of paramagnetic defects in the samples [45]. Compared with Pure TiO_2-X_-550, the resonance signals of the defect-regulated TiO_2-X_ samples are significantly enhanced, suggesting a higher defect concentration.

### 2.2. Analysis of the Photovoltaic Properties

**Figure 5:**
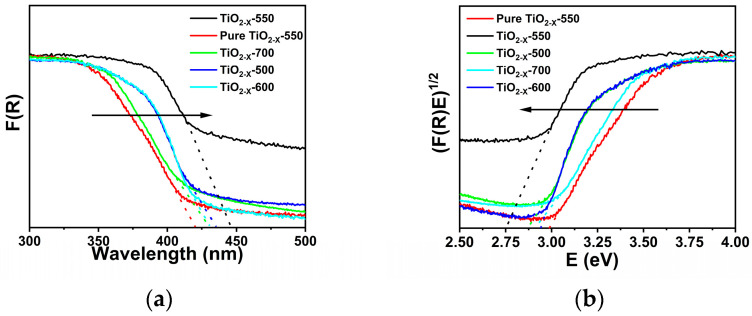
(**a**) UV-vis diffuse reflectance spectra; (**b**) band gap energies of porous TiO_2−X_.

The optical absorption properties of TiO_2-X_-550 were characterized by UV-vis diffuse reflectance spectroscopy (UV-vis DRS) [45]. As presented in Figure 5b, the band gap energy of TiO_2-X_-550 was calculated to be 2.75 eV, which is significantly smaller than that of pristine TiO_2_ (3.2 eV, corresponding to an absorption edge of 387 nm). In addition, extrapolation of the absorption edge in Figure 5a yielded an absorption wavelength of approximately 446 nm, indicating an obvious red shift in the absorption edge. This red shift demonstrates that TiO_2-X_-550 possesses an extended light-response range and enhanced visible-light absorption capability. Similar red-shift behavior was also observed for the samples calcined at other temperatures [45,46,47]. Moreover, the reduced band gap energy and increased specific surface area, especially for TiO_2-X_-550 with a specific surface area of 62.9 m^2^ g^−1^, are beneficial for broadening the spectral response range, enhancing photon utilization, and facilitating mass transfer as well as the migration of reactive species, thus contributing to the improved photocatalytic activity.

**Figure 6:**
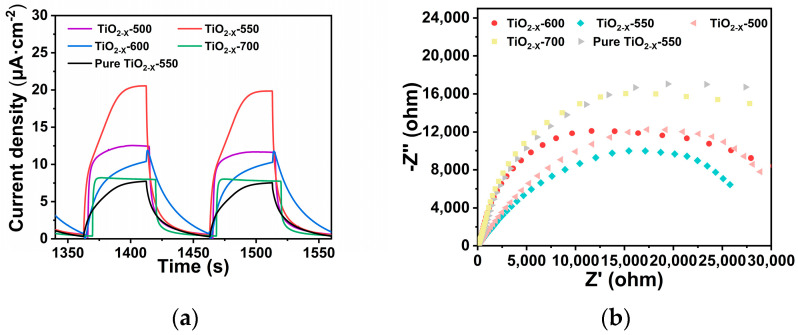
(**a**) Electrochemical photoresponse current test; (**b**) Electrochemical impedance spectrum of porous TiO_2−X_.

From Figure 6a, as shown in the transient photocurrent curves, all samples exhibited obvious and reproducible photocurrent responses under periodic light on–off irradiation, demonstrating that each catalyst could effectively respond to light excitation. Notably, TiO_2-X_-550 showed the highest photocurrent density among all the samples, indicating its superior ability to promote the separation of photogenerated electron–hole pairs and accelerate charge-carrier transport. With the calcination temperature increasing from 500 to 550 °C, the photocurrent response increased markedly. In contrast, further increasing the calcination temperature to 600 and 700 °C led to a gradual decline in photocurrent intensity, suggesting that excessively high calcination temperatures are detrimental to the separation and migration of photogenerated carriers [45,46,48]. According to the EIS Nyquist plots shown in Figure 6b, TiO_2-X_-550 displays the smallest semicircular arc radius, indicating the lowest interfacial charge-transfer resistance and the fastest electron-transfer kinetics among all the samples. By comparison, the samples calcined at higher temperatures exhibit relatively larger impedance arcs, suggesting less efficient interfacial charge transport. This result demonstrates that the introduction of the porous structure in TiO_2-X_-550 can effectively reduce the charge-transfer resistance of photogenerated electrons and thereby promote the photocatalytic reaction [46]. As shown in the Figure S5, all samples exhibit positive slopes in the Mott–Schottky plots, indicating that they are typical n-type semiconductors. Notably, TiO_2-X_-550 shows the smallest slope, suggesting that it has the highest carrier concentration. According to the Mott–Schottky relationship, the slope is inversely proportional to the donor density; therefore, a smaller slope indicates a higher electron donor concentration, which is beneficial for charge transfer and separation, thereby enhancing the photoelectrochemical or photocatalytic performance. In contrast, the other samples exhibit relatively larger slopes, indicating lower carrier concentrations. In addition, the differences in the *x*-axis intercepts among the samples indicate variations in their flat-band potentials, suggesting that the calcination temperature or defect engineering can influence the band structure of the materials. Overall, TiO_2-X_-550, with the smallest slope and the highest carrier concentration, exhibits superior electron transport capability and thus is expected to show better catalytic activity.

**Figure 7:**
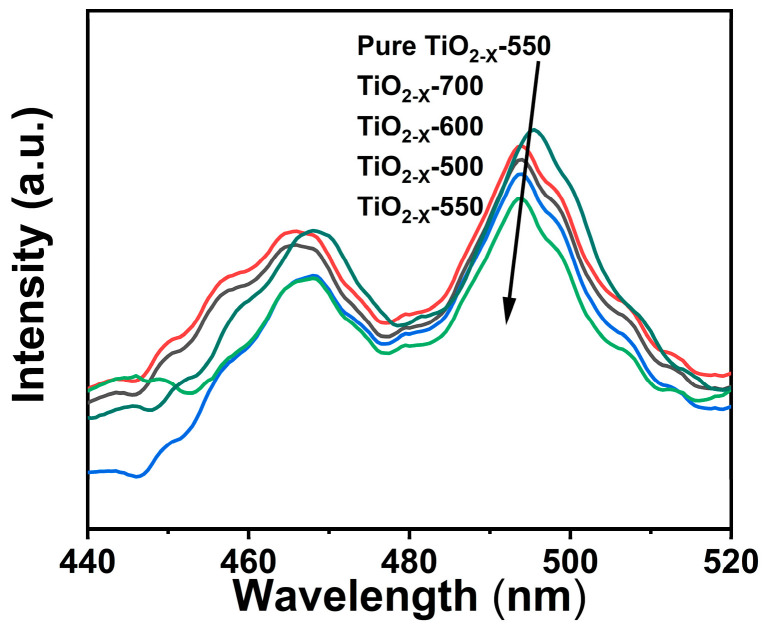
Fluorescence spectra of porous TiO_2−X_.

To further evaluate the separation and recombination behaviors of photogenerated charge carriers, photoluminescence (PL) spectra of the as-prepared samples were recorded. All samples displayed obvious emission signals in the range of 450–510 nm, among which the emission peak at around 495 nm was the most intense. As presented in Figure 7, TiO_2-X_-700 exhibits the highest PL intensity, indicating the most pronounced recombination of photogenerated electron–hole pairs. This finding suggests that the calcination temperature plays a critical role in regulating the electronic structure and surface defect states of TiO_2_. By comparison, TiO_2-X_-550 shows the lowest PL intensity, demonstrating its strongest ability to inhibit charge-carrier recombination and promote the separation of photogenerated electrons and holes, in good agreement with its highest photocatalytic activity [49]. Generally, a lower PL intensity indicates a lower recombination probability of photogenerated carriers and hence a more efficient charge-separation process.

### 2.3. Photocatalytic Performance

**Figure 8:**
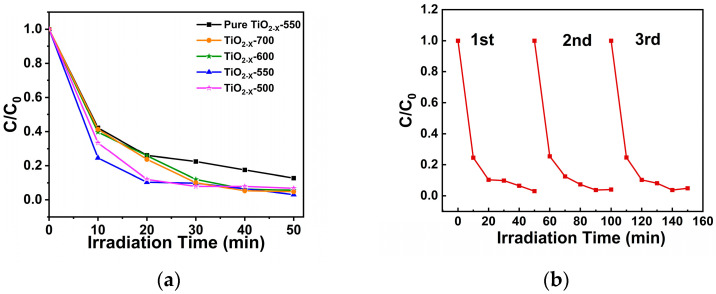
(**a**) Degradation experiments of RhB by porous TiO_2−X_ after calcination at different temperatures; (**b**) Three-cycle degradation experiment of RhB by TiO_2−X_−550.

The photocatalytic performance of the samples was evaluated by degrading RhB (Rhodamine B) solution under simulated sunlight in Figure 8a. Among all the samples, the degradation performance of RhB by the catalysts first increased and then decreased with the increase in calcination temperature. And TiO_2-X_-550 showed the best degradation of RhB, indicating that TiO_2-X_-550 with oxygen vacancies has extremely strong photocatalytic ability. To evaluate the photocatalytic stability of the samples, the prepared samples were used to degrade RhB solution under simulated fluorescent lamps for three cycles. Figure 8b shows that after three cycles, the degradation of RhB by TiO_2-X_-550 remained almost unchanged, and its photocatalytic activity was stable, indicating that the catalytic performance of TiO_2_ with oxygen vacancies has high stability. For further benchmarking, the photocatalytic degradation performance of commercial P25 TiO_2_ was also investigated, and the corresponding results are provided in Figure S6, further demonstrating the superior photocatalytic activity of TiO_2-X_-550. As shown in Figure S7, all samples exhibit good linear relationships between ln(C_0_/C) and irradiation time, indicating that the photocatalytic degradation process obeys pseudo-first-order kinetics. The gradual increase in ln(C_0_/C) with irradiation time further confirms the continuous degradation of the pollutant. Among all samples, TiO_2-X_-550 shows the largest slope, with an apparent rate constant (k) of 0.063 in Figure S7, demonstrating the highest photocatalytic degradation activity.

Superoxide radicals (·O^2−^) and hydroxyl radicals (·OH) are important reactive species in photocatalytic reactions. To further elucidate the reaction mechanism of this system, the roles of these two radicals were investigated. In general, photogenerated electrons can reduce surface-adsorbed oxygen molecules to produce ·O^2−^, whereas photogenerated holes can oxidize water molecules or surface hydroxyl groups to generate ·OH. Their formation processes can be described by the following reaction equations.

TiO_2−X_ + hν → e^−^ + h^+^ (4)

OH^−^ + h^+^ → ·OH (5)

O_2_ + e^−^ → ·O_2_^−^ (6)

·O_2_^−^ + e^−^ + 2H^+^ → H_2_O_2_ (7)

H_2_O_2_ + e^−^ → ·OH + OH^−^ (8)

**Figure 9:**
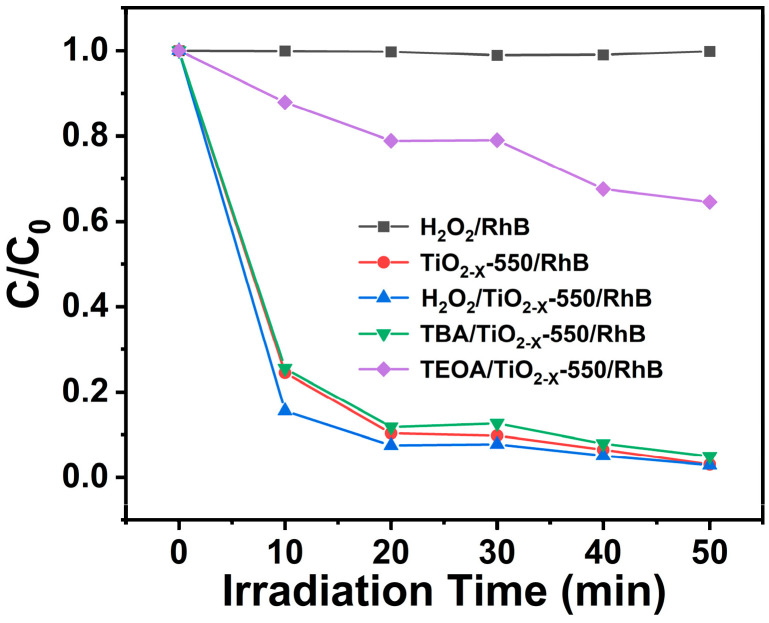
Degradation rate curves of RhB solution under visible light in different photocatalytic degradation systems over the TiO_2-X_-550 photocatalyst.

To further elucidate the photocatalytic degradation mechanism of RhB over TiO_2-X_-550 and identify the predominant reactive species, radical trapping experiments were carried out. In these experiments, tert-butanol (TBA) and triethanolamine (TEOA) were employed as scavengers for ·OH and h^+^, respectively, while H_2_O_2_ was introduced to further evaluate the role of hydroxyl radicals in the degradation process. As illustrated in Figure 9, in the H_2_O_2_/RhB system, the C/C_0_ value of RhB remained close to 1.00 after 50 min of visible-light irradiation, indicating that H_2_O_2_ alone was almost incapable of degrading RhB. In contrast, in the H_2_O_2_/TiO_2-X_-550/RhB system, the C/C_0_ value decreased significantly, showing a much higher degradation efficiency than that of the TiO_2-X_-550/RhB system. This result indicates that the introduction of H_2_O_2_ promoted RhB degradation, suggesting that ·OH participated in the reaction to a certain extent.

After the addition of TBA, the degradation efficiency showed only a slight change compared with that of the TiO_2-X_-550/RhB system, indicating that ·OH was not the primary reactive species. However, upon the addition of TEOA, the degradation of RhB was markedly suppressed, with the C/C_0_ value remaining at 0.64 after 50 min. This result demonstrates that photogenerated holes played a dominant role in the photocatalytic degradation of RhB over TiO_2-X_-550. Therefore, the degradation of RhB mainly proceeded through a hole-dominated photooxidation pathway, while ·OH acted only as a secondary reactive species. The specific process is described as follows:

Photogenerated holes, as the primary reactive species, directly oxidize organic molecules, ultimately yielding oxidation products:

RhB + h^+^ → Oxidation products (9)

In addition, photogenerated holes can first participate in oxidation reactions to generate ·OH, after which ·OH, as a reactive oxygen species, further oxidizes and degrades RhB.

h_vb_ + OH^−^ → ·OH (10)

RhB + ·OH → Oxidation products(11)

**Figure 10:**
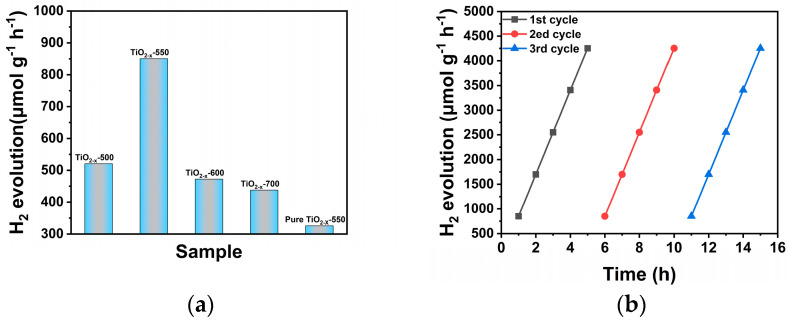
(**a**) Hydrogen evolution of porous TiO_2_ calcined at different temperatures; (**b**) Three−cycle hydrogen evolution of TiO_2−X_−550.

As shown in Figure 10a, the hydrogen production capacity of different samples varies significantly. The TiO_2-X_-550 sample has the highest hydrogen production rate within a unit time, reaching 850.6 μmol·g^−1^·h^−1^, which is much higher than that of TiO_2-X_-500, TiO_2-X_-600, TiO_2-X_-700, and pure TiO_2-X_. The sample synthesized at 550 °C exhibits the optimal photocatalytic hydrogen production activity. This is because an appropriate calcination temperature is conducive to improving the crystal phase, defects, and surface properties of the sample, facilitating the separation and transmission of photogenerated carriers, thereby enhancing the photocatalytic hydrogen production ability. However, excessively high or low calcination temperatures will result in poor crystallization degree or reduced specific surface area, or even excessive agglomeration, which is not conducive to the occurrence of the hydrogen production reaction. At the same time, the appropriate oxygen vacancies or surface defects generated at 550 °C can provide a good transmission path for photogenerated electrons and increase the number of active sites, thus making this sample have a better photocatalytic hydrogen desorption effect. For further benchmarking, the photocatalytic hydrogen evolution performance of commercial P25 TiO_2_ was also evaluated, and the corresponding results are presented in Figure S8, further confirming the superior hydrogen evolution activity of TiO_2-X_-550. As shown in the Figure S8, the H_2_ evolution rate of TiO_2-X_-550 is 850.6 μmol·g^−1^·h^−1^, which is significantly higher than that of commercial P25 TiO_2_ (55 μmol·g^−1^·h^−1^). The hydrogen evolution activity of TiO_2-X_-550 is therefore approximately 15.5 times that of P25, indicating its superior photocatalytic hydrogen production performance. In addition, the wavelength-dependent apparent quantum efficiency (AQE) and apparent quantum yield (AQY) of TiO_2-X_-550 were further evaluated, as shown in Figures S9 and S10. The results show that both AQE and AQY decrease gradually with increasing irradiation wavelength, which is in good agreement with the optical absorption behavior of the catalyst. This observation further confirms the efficient utilization of incident photons by TiO_2-X_-550 and supports its excellent photocatalytic hydrogen evolution performance.

As shown in Figure 10b, in the continuous three-cycle tests, the hydrogen production volume of the samples increased almost linearly with the increase in reaction time. Moreover, the slope of each curve did not change much and the final cumulative hydrogen production volume was similar, indicating that this catalyst can be reused and still perform well in photocatalytic hydrogen production. From the above experiments, it can be concluded that after multiple cycles of reaction, the sample does not show obvious deactivation or decrease in activity, indicating that it has good cycle stability and recyclability in Figures S11 and S12; it also indicates that this substance has good high-temperature resistance and corrosion resistance and is not prone to deactivation. For comparison, the synthetic methods, light sources, catalyst dosages, organic dye degradation efficiencies, and H_2_ evolution rates of representative TiO_2_-based photocatalysts reported in recent studies in Table S4 to illustrate the advantages of our work [50,51,52,53,54]. During the continuous reaction process, it can always retain certain active centers and good interface reaction performance, which has a good guiding significance for its future application in real photocatalytic hydrogen production systems.

### 2.4. Photocatalytic Mechanism

TiO_2-x_-550 exhibits enhanced photocatalytic activity owing to the synergistic effects of oxygen vacancies, Ti^3+^ defect states, and its hierarchical porous framework. Upon light irradiation, TiO_2_ is photoexcited to generate electron–hole pairs. The introduced oxygen vacancies and Ti^3+^ species act as electron-trapping sites or shallow donor levels, which facilitate charge separation, suppress electron–hole recombination, and simultaneously broaden the light absorption range as well as promote the activation of adsorbed oxygen molecules. In addition, the hierarchical meso-/macroporous channels favor the diffusion and transport of reactant molecules into the inner surface of the photocatalyst, while the thin framework shortens the migration distance of photogenerated charge carriers, thereby improving interfacial charge transfer efficiency. During the photocatalytic process, photogenerated holes react with adsorbed H_2_O or OH^−^ to produce highly oxidative hydroxyl radicals, whereas photogenerated electrons reduce adsorbed O_2_ to superoxide radicals, both of which contribute to the degradation of organic pollutants. Meanwhile, part of the conduction-band electrons participate in proton reduction to generate hydrogen, The detailed reaction process is shown in Equations (12)–(15) [55,56,57]. Therefore, the improved photocatalytic degradation and hydrogen evolution performance of TiO_2-x_-550 can be attributed to the combined contributions of defect-induced electronic modulation, enhanced visible-light utilization, accelerated mass transport, and efficient charge separation and migration.

TiO_2−X_ + hν → e^−^ + h^+^ (12)

H_2_O/OH^−^ + h^+^ → ·OH (13)

2H^+^ + 2e^−^ → H_2_ (14)

O_2_ + e^−^ → O_2_^·−^ (15)

## 3. Conclusions

In summary, a hierarchical porous TiO_2_ photocatalyst rich in oxygen vacancies was successfully prepared using IRA-900 as a template, forming a three-dimensional interconnected pore structure. It exhibited excellent activity in RhB and mixed-dye degradation, with over 85% RhB removed within 50 min, and a photocatalytic hydrogen evolution rate of 850.6 μmol·g^−1^ h^−1^. The performance enhancement mainly arises from the synergy between the hierarchical porous structure and oxygen vacancies: the former improves mass transfer and provides abundant active sites, while the latter tunes the electronic structure and promotes charge separation. Overall, this strategy offers an effective route to enhance the photoelectric conversion performance of TiO_2_ and provides theoretical and experimental support for defect engineering and structural design of TiO_2_-based photocatalysts; it also shows great potential for photocatalytic hydrogen evolution applications.

## 4. Materials and Methods

### 4.1. Materials

No further purification of chemicals before experiments. Tetrabutyl titanate (C_16_H_36_O_4_Ti) was purchased from Shanghai Boer Chemical Co., Ltd. (Shanghai, China). Acetic acid (C_2_H_4_O_2_) and absolute ethanol (C_2_H_6_O) were supplied by Tianjin Tianli Chemical Co., Ltd. (Tianjin, China). Hydrochloric acid (HCl) was obtained from Sinopharm Chemical Reagent Co., Ltd. (Shanghai, China). IRA-900 purchased from Alfa Aesar Co., Ltd. (Shanghai, China).

### 4.2. Preparation of Micron Porous TiO_2-X_

First, 10 mL of tetrabutyl titanate, 5 mL of acetic acid, and 40 mL of absolute ethanol were mixed and stirred at 540 rpm for 5 min. Subsequently, a mixed solution containing 10 mL of deionized water, 10 mL of absolute ethanol, and 2 mL of hydrochloric acid was added dropwise under continuous stirring. After further stirring for 5 h, a homogeneous TiO_2_ sol was obtained. Then, 9.0 g of IRA-900 anion-exchange resin was introduced into the TiO_2_ sol, and the mixture was stirred at 40 °C for 20 h to ensure sufficient impregnation of the resin by the sol. The impregnated resin and residual sol were subsequently transferred into a Teflon-lined stainless-steel autoclave and aged statically at 60 °C for 24 h, during which the TiO_2_ sol further evolved into a gel precursor. The obtained product was collected by separation, thoroughly washed, and dried to afford the IRA-900-supported TiO_2_ gel precursor. The resulting gel precursor was pre-calcined in a tubular furnace under a mixed atmosphere of 2% H_2_ and 98% inert gas. The pre-calcined sample was then transferred to a muffle furnace and heated at a rate of 1.5 °C min^−1^ to 500, 550, 600, or 700 °C, respectively, and maintained at the target temperature for 360 min. The obtained micron-sized porous TiO_2_ microspheres gels were denoted as TiO_2−X_-500, TiO_2-X_-550, TiO_2-X_-600, and TiO_2-X_-700, respectively. Among them, the template removal efficiency in the repeated experiments was 90.7% ± 2.5%.

### 4.3. Characteristic

The morphology and microstructure of the samples were characterized by SEM (TESCAN MIRA LMS, Brno, Czech Republic, 5 kV) and TEM (FEI Talos F200X G2 AEMC, Thermo Fisher Scientific, Waltham, MA, USA, 120 kV). The crystal structures were analyzed by XRD (Shimadzu XRD-6100, Shimadzu Corp., Kyoto, Japan), and the average crystallite size was calculated using the Scherrer equation. FT-IR spectra were recorded on a Spectrum 400 spectrometer (PerkinElmer, Waltham, MA, USA) in the range of 4000–500 cm^−1^. The specific surface area and pore structure were determined by N_2_ adsorption–desorption measurements on an ASAP 2460 analyzer (Micromeritics Instrument Corp., Norcross, GA, USA), and the pore size distribution was calculated using the BJH model. UV-vis DRS spectra were obtained using a Shimadzu UV-2450 spectrophotometer (Shimadzu Corp., Kyoto, Japan) equipped with an integrating sphere, with BaSO_4_ as the reference. XPS analysis was performed on an ESCA PHI 5000 instrument (Physical Electronics Inc., Chanhassen, MN, USA). PL spectra were collected on a PerkinElmer LS 55 fluorescence spectrometer (PerkinElmer, Waltham, MA, USA). Photoelectrochemical measurements were carried out in a three-electrode system with platinum wire as the counter electrode, Ag/AgCl as the reference electrode, and sample-coated conductive glass as the working electrode. The photocurrent response was measured in 2 M Na_2_SO_4_ electrolyte under irradiation from a 300 W xenon lamp (SAN-EI Electric Co., Ltd., Osaka, Japan), and the transient photocurrent at zero bias was recorded on a CHI 660E electrochemical workstation (CH Instruments, Inc., Austin, TX, USA). EIS measurements were performed over a frequency range of 10^−2^–10^−5^ Hz.

### 4.4. Catalytic Process

The photocatalytic activity was assessed by monitoring the degradation of rhodamine B (RhB,10 mg·L^−1^) under simulated solar-light irradiation. A 300 W xenon lamp was used as the irradiation source. Before illumination, 0.05 g of photocatalyst was dispersed in 50 mL of RhB aqueous solution and stirred in the dark for 30 min, followed by ultrasonication for 10 min, to achieve adsorption–desorption equilibrium between the catalyst surface and the dye molecules. During the photocatalytic reaction, aliquots were collected every 10 min and centrifuged to remove the suspended catalyst particles. The absorbance of the resulting supernatant was measured at 554 nm with a UV-vis spectrophotometer, and the corresponding RhB concentration was determined using a standard calibration curve. The photocatalytic degradation efficiency was then calculated accordingly.

The photocatalytic performance was evaluated by monitoring the hydrogen evolution rate. The photocatalytic hydrogen evolution performance of TiO_2_ for water splitting was evaluated as follows. First, 0.5 g of the sample was accurately weighed and placed into a quartz reactor. Then, 300 mL of deionized water and 30 mL of methanol were added to form the reaction solution. Methanol was employed as a sacrificial agent to effectively consume photogenerated holes, suppress electron–hole recombination, and thus enhance the hydrogen evolution reaction. Prior to irradiation, the suspension was ultrasonically dispersed for 30 min to ensure the uniform distribution of the catalyst particles in the reaction medium. Subsequently, the reactor was evacuated and purged with nitrogen to remove residual air and dissolved oxygen, thereby ensuring an oxygen-free and closed reaction environment. After the pretreatment, a xenon lamp was used as the simulated light source to initiate the photocatalytic water-splitting reaction. Irradiation was carried out using optical filters in the ranges of 310–400 nm and 400–700 nm, respectively. During the reaction, gas samples were collected every 1 h, and the amount of hydrogen evolved was quantified by gas chromatography. The photocatalytic activity and hydrogen evolution performance of the TiO_2_ samples were finally evaluated according to the hydrogen production rate and its variation as a function of reaction time.

## Data Availability

Data supporting the findings of this study are not currently publicly available owing to related ongoing research but can be requested from the corresponding author.

## Supplementary Materials

The following supporting information can be downloaded at: https://www.mdpi.com/article/10.3390/gels12050370/s1. Figure S1: EDAX spectrum and elemental composition of TiO_2-X_-550; Figure S2: Micro-CT image of the TiO_2-X_-550; Figure S3: XPS survey spectrum of TiO_2-X_-550; Figure S4: EPR of porous TiO_2-X_; Figure S5: Mott–Schottky plots of TiO_2-X_ samples prepared at different calcination temperatures; Figure S6: Comparison of the photocatalytic degradation performance of different TiO_2_ samples; Figure S7: Pseudo-first-order kinetic plots of photocatalytic degradation over TiO_2_ samples calcined at different temperatures; Figure S8: Comparison of the hydrogen evolution performance of different TiO_2_ samples; Figure S9: Apparent quantum efficiency (AQE) of TiO_2-X_-550; Figure S10: Apparent quantum yield (AQY) of TiO_2-X_-550 at different wavelengths; Figure S11: XRD of TiO_2-X_-550 before and after three cycles hydrogen evolution; Figure S12: FITR of TiO_2-X_-550 before and after three cycles hydrogen evolution; Table S1: XRD peak parameters and calculated crystallite sizes of the TiO_2-X_-550; Table S2: Nitrogen adsorption–desorption data of porous TiO_2-X_ at different calcination temperatures; Table S3: Micro-CT-derived pore structure parameters of the TiO_2-X_-550; Table S4: Comparison of synthesis methods, reaction conditions, and photocatalytic performance of representative TiO_2_-based photocatalysts reported in previous studies. References [50,51,52,53,54] are cited in the Supplementary Materials.
