# Supplementary material for: Oxygen-Deficient TiO2 Aerogel for Enhanced Photocatalytic Performance
Source: Gels. 2026 Apr 28;12(5):370. doi: 10.3390/gels12050370 (PMC13206627; doi:10.3390/gels12050370)
Supplement: Supplementary file 1 [file gels-12-00370-s001.zip › gels-4251107-supplementary.pdf]

Supplementary Information

Oxygen-Deficient TiO_2 Aerogel for Enhanced Photo-catalytic Performance

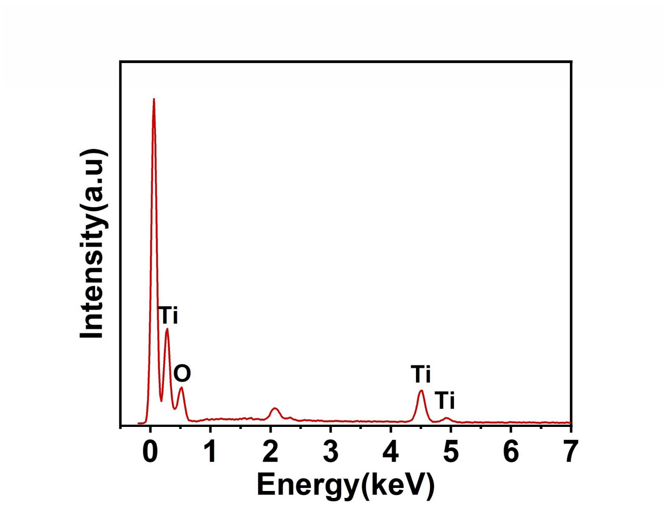

Figure S1. EDAX spectrum and elemental composition of TiO_{2-x}-550

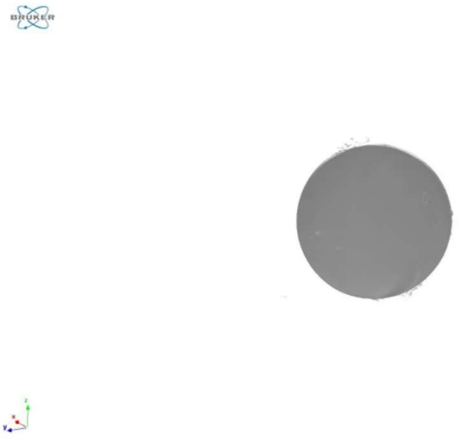

Figure S2. Micro-CT image of the TiO_{2-x}−550

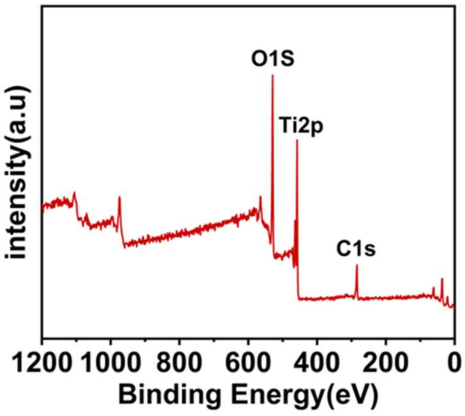

Figure S3. XPS survey spectrum of TiO_{2-x}–550

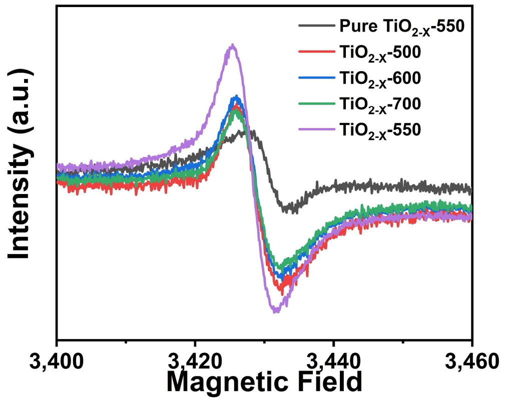

Figure S4. EPR of porous TiO_{2-x}.

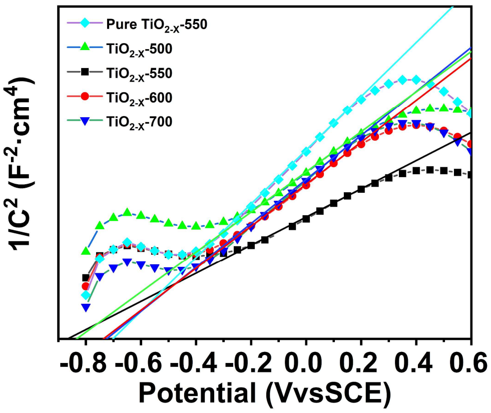

Figure S5. Mott−Schottky plots of TiO_{2-X} samples prepared at different calcination temperatures.

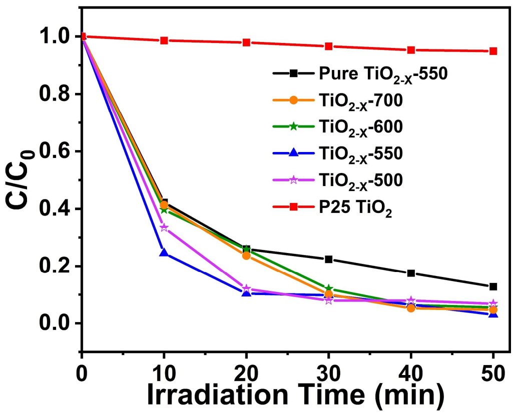

Figure S6. Comparison of the photocatalytic degradation performance of different TiO_2 samples.

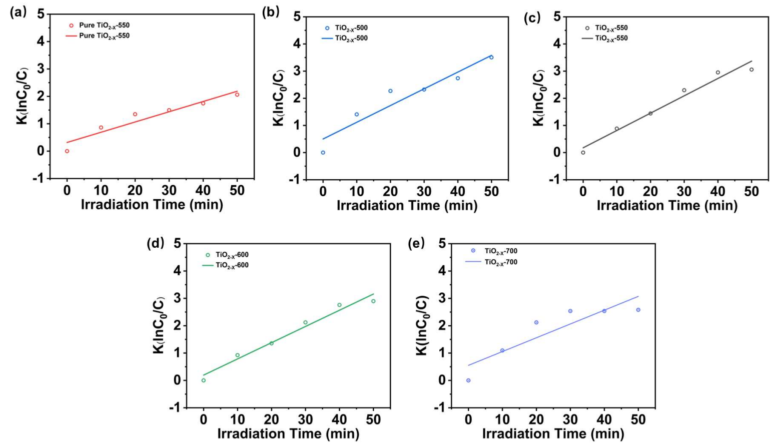

Figure S7. Pseudo−first−order kinetic plots of photocatalytic degradation over TiO_2 samples calcined at different temperatures.

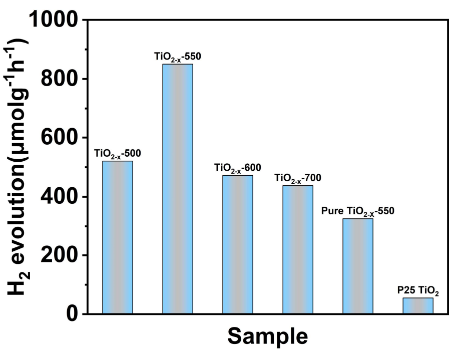

Figure S8. Comparison of the hydrogen evolution performance of different TiO_2 samples

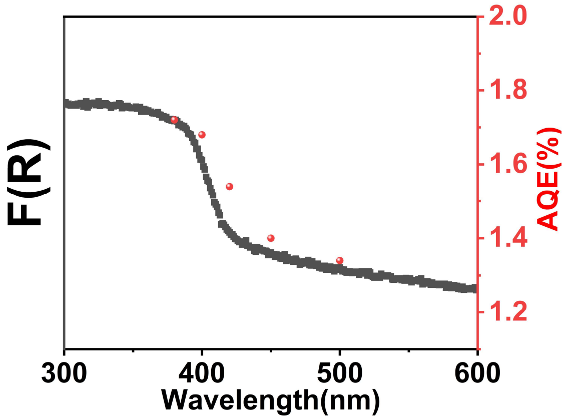

Figure S9. Apparent quantum efficiency (AQE) of TiO_{2-x}–550

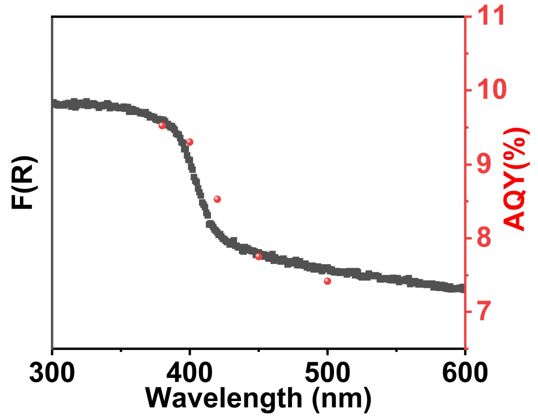

Figure S10. Apparent quantum yield (AQY) of TiO_{2-x}–550 at different wavelengths

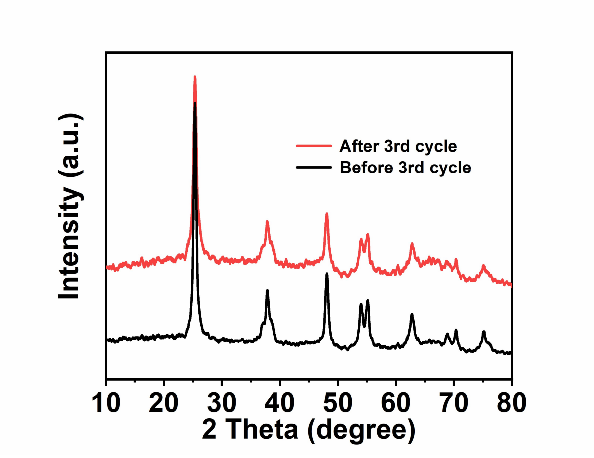

Figure S11. XRD of TiO_{2-x}–550 before and after three cycles hydrogen evolution.

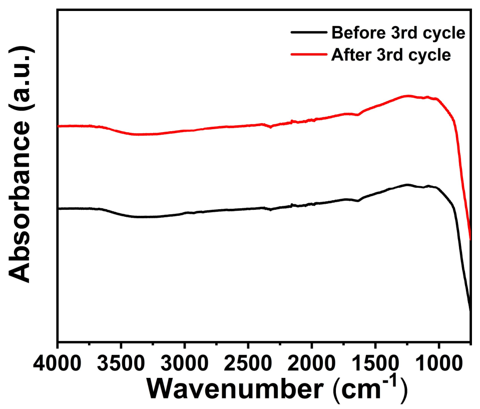

Figure S12. FITR of TiO_{2-x}–550 before and after three cycles hydrogen evolution.

Table S1. XRD peak parameters and calculated crystallite sizes of the TiO_{2-x}−550

Crystallite patterns	A(101)	A(004)	A(200)	A(105)	A(201)	A(204)	A(116)
**2θ/°**	25.3	37.8	48.0	53.9	54.9	62.9	68.8
**β**	0.48	1.07	0.62	0.98	0.74	0.92	0.98
**Crystallite size /nm**	16.7	7.7	13.9	9.0	12.0	10.1	9.7

Table S2 Nitrogen adsorption-desorption data of porous TiO_{2-x} at different calcination temperatures.

**Sample**	**Pure TiO_2-550**	**TiO_{2-x}-500**	**TiO_{2-x}-550**	**TiO_{2-x}-600**	**TiO_{2-x}-700**
**S_{BET}(m^2 g^{-1})**	14.4	42.2	62.9	41.4	19.2
**Pore Volume (cm^3 g^{-1})**	0.05	0.12	0.22	0.16	0.05
**Average pore size (nm)**	14.78	25.7	33.48	23.78	11.75

Table S3 Micro-CT-derived pore structure parameters of the TiO_{2-x}–550.

Parameter	Value	Unit
**Total porosity**	46.26	%
**Open porosity**	44.95	%
**Closed porosity**	2.37	%
**Total pore volume**	1.78×10^8	μm^3
**Open pore volume**	1.73×10^8	μm^3
**Closed pore volume**	5.03×10^6	μm^3
**Connectivity**	171118	--
**Connectivity density**	4.44×10^{-4}	μm^{-3}
**Pixel size**	0.65	μm

Table S4 Comparison of synthesis methods, reaction conditions, and photocatalytic performance of representative TiO_2–based photocatalysts reported in previous studies

Potocatalyst	Synthesis metheod	Light source	Catalyst Dosage	organic dye degradation efficiency	H_2 evolution Rate(μmol·g^{-1}·h^{-1})	reference
TiO_{2-X}	High-temperature chemical reduction method	300 W Xe lamp	10 mg/L	94%	523.73	1
M-TiO_2-2	wet impregnation immobilization method	300 W Xe/Hg lamp	0.5 g/L	-	502.2	2
CTR2	hydrothermal synthesis	30W LED light	0.25 g/L	95%	950	3
4wt% Ni_3（HITP）$_2$/$Ti_{1-x}O_2$-2	hydrothermal synthesis	300 W Xe lamp	0.2 g/L	-	3520	4
SiC/B–TiO_2	hydrothermal synthesis	LED light source	-	-	1350	5

Reference:

[1] Zhang, B.; Wang, D.; Cao, J.; He, W.; Liu, G.; Liu, D.; Zhao, C.; Pan, J.; Liu, S.; Zhang, W.; Fang, X.; Zhao, L.; Wang, J. Tuning Stark effect by defect engineering on black titanium dioxide mesoporous spheres for enhanced hydrogen evolution. Chin. Chem. Lett. 2024, 35(11), 110254. https://doi.org/10.1016/j.cclet.2024.110254.

[2] Menin, J.; Cechin, C. N.; Burrow, R. A.; Iglesias, B. A.; Daudt, N. F.; Ledesma, G. N.; Cargnelutti, R.; dos Santos, S. S.; Lang, E. S.; Tirloni, B. Solar-driven hydrogen production by a modified titania matrix with Pd complexes as co-catalysts. J. Mol. Struct. 2026, 1349, 143645. https://doi.org/10.1016/j.molstruc.2025.143645.

[3] Banerjee, N.; Roy, A.; Nair, R. G. Z-Scheme modulated direct-direct Titania-CdS heterojunctions: A strategy-driven approach to enhance photoelectrochemical and solar photocatalytic hydrogen evolution. *Int. J. Hydrogen Energy* **2025**, 173, 151332. https://doi.org/10.1016/j.ijhydene.2025.151332.

[4] Guo, X.; Zhang, Z.; Li, K.; Liu, Y.; Zhang, X.; Xu, L.; Li, B. Conductive MOF/defect titanium dioxide S-scheme heterojunction with enhanced charge transfer for efficient photocatalytic hydrogen generation. *Int. J. Hydrogen Energy* **2025**, 128, 665-673. https://doi.org/10.1016/j.ijhydene.2025.04.032.

[5] Niero, A. L. de S.; Pasini, S. M.; Daguer, P. H.; Sica, E. T.; Oechsler, B. F.; González, S. Y. G.; Hotza, D. Structured macroporous brown TiO_2-SiC catalysts for ethanol-water photoreforming under visible light for hydrogen production. *Int. J. Hydrogen Energy* **2025**, 140, 26-35. https://doi.org/10.1016/j.ijhydene.2025.05.273.
